# Development of a novel swine model carrying humanized *APP* and *PSEN1*

**DOI:** 10.1002/alz.089607

**Published:** 2025-01-03

**Authors:** Junchul Yoon, Kaylynn Monarch, Emily Kiesewetter, Katherine Rodriguez‐Lukey, Sehwon Koh, Kyungjun Uh, Randall S Prather, Tim Jarome, Tim Allen, Kiho S Lee

**Affiliations:** ^1^ University of Missouri, Columbia, MO USA; ^2^ Futuristic Animal Resource & Research Center (FARRC), Cheongju, Chungcheongbuk‐do Korea, Republic of (South); ^3^ Virginia Polytechnic Institute and State University, Blacksburg, VA USA; ^4^ Florida International University, Miami, FL USA

## Abstract

**Background:**

Preclinical animal models are essential for the development of effective treatments. For instance, the 5xFAD mouse model successfully represents the pathophysiology of Alzheimer’s disease (AD). Expression of humanized *APP* (K670N/M671L ‐ Swedish, I716V ‐ Florida, V717I ‐ London) and *PSEN1* (M146L and L286V), found in early onset AD patients, induces the production of amyloid‐β 42 (Aβ42) and amyloid deposition, gliosis, and progressive neuronal loss. While these mouse models are necessary to identify mechanisms of the disease progression, translating the findings by using large animal models such as pigs allows us to explore treatments under clinical conditions, and therefore, improve the success of clinical trial outcomes. For example, the gray‐to‐white matter ratio and the complexity of the distribution of crossing fibers in the pig brain is more similar to humans than are rodents.

**Method:**

Phenotypes of previous swine models carrying humanized *APP* and *PSEN1* to mimic the 5xFAD mouse model did not align with the mouse model, presumably due to differences in aging rates or variation in the expression level of the genes. To accelerate the impact of the humanized *APP* and *PSEN1*, we first inactivated endogenous porcine *APP* and *PSEN1* genes by using the CRISPR/Cas9 system in fetal fibroblast cells. Subsequently, constructs designed to express human *APP* and *PSEN1* under the control of the neuron specific *Thy1* promoter were transfected into the cells. Cells carrying the humanized *APP* and *PSEN1* genes and the cells were used for somatic cell nuclear transfer to produce AD swine model.

**Result:**

Thirteen piglets were born from a single pregnant sow and the genotyping of the piglets indicated that the piglets carried inactivated porcine *APP* and *PSEN1* and carry humanized *APP/PSEN1* as expected. Immunohistochemistry on the brain of the newborn AD piglets revealed an elevated abundance of Aβ42 compared to the wild type.

**Conclusion:**

Production of the novel AD swine model will offer a new pre‐clinical animal resource to expand our understanding of Alzheimer’s disease pathogenesis and develop effective treatments against the disease.

